# Notes and News

**Published:** 1899-08-26

**Authors:** 


### Notes and News.

(Collected and Communicated.)

A considerable amount of typhoid is present in Paris. It has been prevalent during the last two months, and the water supply is credited with being the cause. The Seine is now very low from the long drought.

It is reported that ankylostomiasis, the so-called tunnel anæmia or miner's chlorosis, which proved such a trouble during the making of the Suez Canal and the St. Gothard Tunnel, has become very prevalent among the coal miners in the Liege district in Belgium.

Great complaints are being made on every hand about the glycerinated calf lymph which is being supplied by the Local Government Board, and assertions are being broadly made that, in consequence of the great demand for it, the Government lymph is being "watered down." It is a great pity, for not only is it a serious tax upon public vaccinators to have to obtain an independent supply, but the whole affair tends to bring the operation into contempt. The antivacs are far too astute not to demand Government vaccine, which, under the provisions of the Act, they have the right to do, especially when they see that, under present circumstances, as they would say, "it can do no harm." Meanwhile the public vaccinator has to make waste journeys and gets no fee..

News from Oporto shows that plague still continues in that city, and it is stated that it is intended to establish that futile thing a sanitary cordon round the town. So far, it is said that none of the English colony there have been attacked. As showing how persistently both the officials and the people in some countries persist in trying to hush up all ill tidings regarding epidemic disease, it is worth noting that Dr. Jorge advised the Government as long ago as July 12th that the cases presented all the appearances of plague, and on the 28th confirmed the existence of the disease. Yet the Portuguese Government did not admit the existence of the epidemic until August 14th. It should be added that Dr. Jorge has been rewarded by receiving over 50 threatening letters, and that various hostile demonstrations have been made against him, making it necessary that he should be furnished with police protection. Meanwhile, the epidemic spreads; the latest news being that fresh cases have been discovered, all of them acute.

A meeting of the Russian Famine Relief Fund will be held on August 30th, at four p.m., in the Pompadour Room at the Hotel Cecil. The Rev. A. Francis, chairman of the committee in St. Petersburg, will preside, and will describe his experience in the famine-stricken districts and the method of distributing relief.

Owing to the want of frankness on the part of officials in Portugal respecting the plague, suspicions have been aroused in other countries, and searching inquiries have been made, with the result that reports have been spread that the plague has entered Italy and Russia. Siberian plague is said to have broken out at Samora, and one town in South-eastern Russia has been isolated. Traffic is restricted on the Volga.

According to a telegram received from Major Donald Ross the expedition sent out by the Liverpool School of Tropical Medicine has succeeded in discovering the species of mosquito concerned in the propagation of malaria in the Sierra Leone district. It is believed by the Liverpool school that the Government will now send out representative medical men to assist their own in the further prosecution of the researches.

The following members of the medical profession have accepted the invitation of the committee of management of the Seamen's Hospital Society to join the teaching staff of the London School of Tropical Medicine: E. Treacher Collins, Esq., F.R.C.S., Malcolm Morris, Esq., F.R.C.S., L. W. Sambon, Esq., M.D. (Naples), and Professor W. J. Simpson, M.D., F.R.C.P., D.P.H.

The useful work already being done by the very recently instituted Liverpool School for Tropical Diseases should serve to stimulate the speedy establishment of the intended school for the study in the same field of medicine to be worked in connection with the Seamen's Hospital at their branch institution at the Victoria and Albert Docks. It is intended that the Colonial Medical Service cadets shall pass through the school, and it will be open to other students as well. It will be of untold value to medical missionaries and all those who have to carry on work in the tropics, where up to the present time no adequate knowledge of local diseases has been obtainable. It is necessary to raise a capital sum of £11,500, and an income of £2,000, to enable the work to be carried on.

The following interesting surgical performances have found their way into the lay press, and have emanated from New York: A man was stabbed to the heart, the ribs being parted and the heart exposed. The "physicians" injected saline solution to replace the blood between the pulsations, and one of them rapidly passed a needle and thread through the left ventricle, and "took several stitches." The patient rallied, and when the report was issued belief in his recovery is stated. The other case is that of a millionaire, who was suffering from an abdominal ailment, the nature of which was diagnosed by the patient swallowing an india-rubber bulb, covered with photographic film. When the bulb reached the stomach it was inflated. An X-ray exposure was then made, the bulb deflated, and then withdrawn. The result was an excellent photograph of the interior of the stomach revealing a large tumour. The name of the patient is given.

